# N-Substituted Auxiliaries for Aerobic Dehydrogenation of Tetrahydro-isoquinoline: A Theory-Guided Photo-Catalytic Design

**DOI:** 10.1038/s41598-019-47735-y

**Published:** 2019-08-02

**Authors:** Savithra Jayaraj, Abraham K. Badu-Tawiah

**Affiliations:** Department of Chemistry and Biochemistry, The Ohio State University, Columbus, OH 43210 USA

**Keywords:** Mass spectrometry, Catalytic mechanisms

## Abstract

Visible-light mediated aerobic dehydrogenation of N-heterocyclic compounds is a reaction with enormous potential for application. Herein, we report the first complete aerobic dehydrogenation pathway to large-scale production of isoquinolines. The discovery of this visible light photoredox reaction was enabled through the combination of mathematical simulations and real-time quantitative mass spectrometry screening. The theoretical calculations showed that hyper-conjugation, the main underlying factor hindering the aerobic oxidation of tetrahydroisoquinolines, is relieved both by π- and σ-donating substituents. This mechanistic insight provided a novel photocatalytic route based on N-substituted auxiliaries that facilitated the conversion of tetrahydroisoquinolines into the corresponding isoquinolines in just three simple steps (yield 71.7% in bulk-solution phase), using unmodified Ru(bpy)_3_Cl_2_ photocatalyst, sun energy, atmospheric O_2_, and at ambient temperature.

## Introduction

Motivated by interests in green chemistry, current catalytic methods strive to achieve high-yields of target compounds while avoiding the production of wastes^1,2^. The development of new catalysts for the dehydrogenation of tetrahydroquinolines (THQ) and tetrahydroisoquinolines (THiQ) has attracted much attention because this transformation represents one of the most atom-efficient means to produce quinolines and isoquinolines that are an integral part of numerous pharmaceuticals and bioactive molecules^3–6^. The use of oxygen as oxidant is a recent advancement^7^, but aerobic oxidation of THiQ is often incomplete, and the reasons for this peculiar reactivity are incompletely understood.

Although impressive insights have been gained from theoretical calculations, realistic screening methods needed to verify theoretical finds are lacking. Instead, findings from calculations are most often investigated using gas-phase or reduced pressure experiments in which the generated bare transition-metal ions/complexes typically have oxidation states that are unusual in condensed-phase chemistry^8–10^. Current traditional condensed-phase screening methods employ batch-mode operation to optimize condensed-phase photoreaction conditions, including the proper selection of catalysts and solvent/reagent. Although this is a versatile technique, it suffers from poor light penetration, long diffusion distances, and large reagent/substrate consumption^11^. Newer techniques such as multi-dimensional reaction screening microfluidic platforms^11–14^, microchip reactors^15,16^ and the use of micro-emulsions^17,18^ as reactors have overcome some of the barriers in the traditional batch-mode processing. Unfortunately, these micro-reactor based techniques still require (i) at least milligram (micromole) quantities of catalyst/substrates, which may be exorbitantly large for initial reaction screening, and (ii) separate workout procedures for evaluation of reaction progress.

To contribute to the emerging field of accelerated droplet reaction screening^19–22^, the current study applies a theory-guided photoreaction screening approach to investigate the underlying mechanistic difference between the reactivity of THQ and THiQ. The ultimate objective is to identify green catalytic conditions that are directly transferable to bulk solution-phase for large scale synthesis. Not surprisingly, the chemical relationship between quinolines and isoquinolines, as well as their uniqueness, have been apparent since the beginning of physical organic chemistry^23,24^. What is surprising is that until recently, the conversion of THQ/THiQ to quinolines/isoquinolines is still practiced most often with stoichiometric metal oxidants such as MnO_2_^25–27^ and IBX (o-iodobenzoic acid)^28^. One of the most successful modern catalytic methods for general dehydrogenation reactions rely on transition metal complexes mainly of pincer ligands^29,30^. In spite of the recent progress with iridium-based catalysts^30^, the development of methods that involve the use of milder reaction conditions (e.g., ambient temperature) and that which utilizes more abundant, inexpensive, and renewable reagents for the dehydrogenation of N-heterocycles is highly desirable. In this regard, iron pincer complexes have been proposed^31^. Although promising, dehydrogenation with iron pincer catalysts requires long reaction times (~30 h) owing to the reversible nature of the process (i.e., iron pincer complexes catalyzes both dehydrogenation and hydrogenation, making the desired dehydrogenation reaction difficult in the presence of H_2_ byproducts). Aerobic oxidative dehydrogenation represents an emerging cleaner alternative in which molecular oxygen (or air) can be used as a cheaper and less polluting stoichiometric oxidant^7,31–34^. Here too, reaction systems utilizing transition metal catalysts require long reaction times and high temperatures. Visible light-mediated photo-redox dehydrogenation reactions have also been reported that are based on the use of air^35^ or external oxidants (e.g., BrCCl_3_^36,37^). *In all cases*, *however*, *aerobic oxidative dehydrogenation of THiQ resulted in the formation of the imine intermediate*.

**Figure 1:**
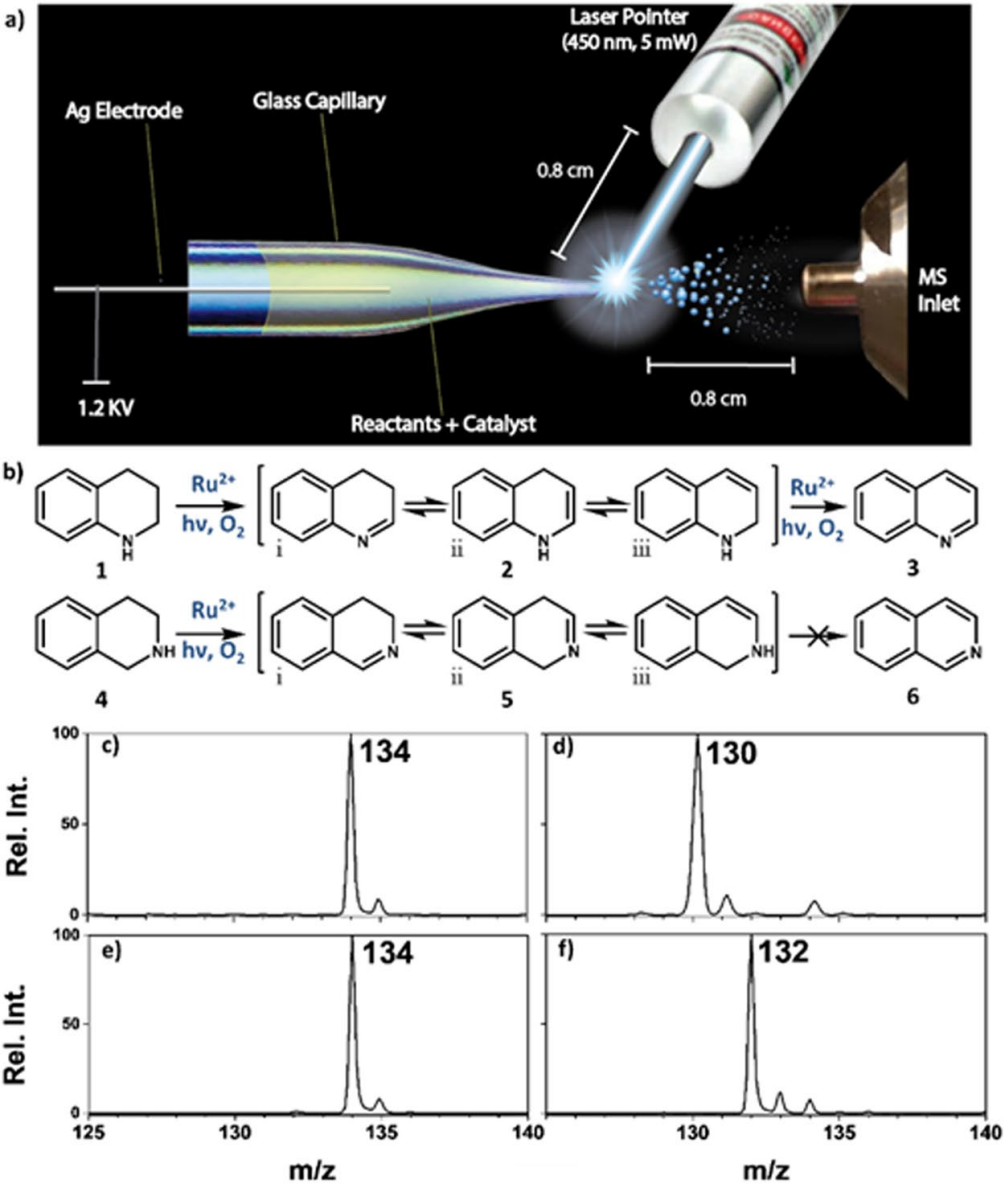
(**a**) Schematic of nESI mass spectrometry based real-time photoreaction screening platform, fitted with blue laser source (450 nm, 5 mW). (**b**) Schematic illustrating dehydrogenation of THQ (**1**, *MW* 133) via dihydroquinoline (**2**, *MW* 131), which further oxidizes to give quinoline (**3**, *MW* 129) while the corresponding isomer THiQ (**4**, *MW* 133) produces only the dihydrosioquinoline (**5**, *MW* 131) as the main product. Dehydrogenation of 100 µM THQ with 5 µM of Ru(bpy)_3_^2+^ in ACN using the real-time photoreaction screening platform: mass spectra showing analysis when (**c**) visible light is off, (**d**) after 2 minutes of visible light exposure; the dehydrogenation of 100 µM THiQ with 5 µM of Ru(bpy)_3_^2+^ in ACN was also performed and the recorded mass spectra are provided when (**e**) visible light is off and (**f**) after 2 minutes of laser irradiation.

Our previous work focused on synthesis of quinolines from THQs^38^. Owing to the fact that the isoquinoline moiety is more common in pharmaceutical compounds (due to its stability^25^) than the quinoline counterpart, we sought to develop a much cleaner and milder photocatalytic strategy based on the use of safe solar photons, ambient air and temperature. Therefore, we used a pico-mole scale reaction screening platform to study the mechanism governing the aerobic oxidation of THiQ under a small-bulk droplet-based experimental conditions. The aerobic dehydrogenation reaction was initiated by the commercially available visible-light-harvesting complex [Ru(bpy)_3_]Cl_2_ (bpy = 2,2′-bipyridine). We combined real-time ambient mass spectrometry (MS) reaction screening and density functional theory (DFT) calculations to determine, and subsequently rationalize, the factors limiting the complete oxidation of THiQ. We carried out our studies using the photoreaction screening platform shown in Fig. 1a, which consisted of a nano-electrospray ionization (nESI) source fitted with a portable laser source (450 nm, 5 mW). By examining the photoreaction products/intermediates contained in the ensuing electrosprayed charged microdroplets with a mass spectrometer, we sought to establish synthesis methodologies for complete aerobic oxidation of THiQ that is based on manipulation of electronic influence of N-substitutions. DFT calculations identified hyper-conjugation as the source of the reactivity bottleneck in THiQ, relieved both by *pi* and *sigma* donating substituents. A comparison of this theoretical insight and real-time experimental results, in turn, enabled us to rationally design a novel bulk-phase photocatalytic strategy based on three simple steps: (i) N-substitution of a suitable auxiliary to reduce hyper-conjugation, (ii) photoredox oxidation of the N-substituted THiQ using Ru(bpy)_3_Cl_2_, and (iii) removal of N-substituted auxiliary to generate isoquinolines.

## Results and Discussion

### Real-time photoreaction screening platform

To achieve real-time photoreaction screening, the amine reactant (i.e., THQ or THiQ) and Ru(bpy)_3_Cl_2_ photocatalyst were pre-mixed and contained in the glass capillary of the nESI emitter. Application of direct current (DC) voltage (1.2 kV) to the amine/Ru(bpy)_3_Cl_2_ mixture results in the generation of electrosprayed charged droplets that subsequently transfer protonated amine and Ru(bpy)_3_Cl_2_ species to the mass spectrometer for characterization. In the presence of applied visible light excited-state chemical reactions can be performed in the electrospray environment where the initial interaction of the amine and the excited Ru*(bpy)_3_^2+^ photocatalyst results in the formation of radical cations and reduced ground state Ru(bpy)_3_^+^ species. Regeneration of the original Ru(bpy)_3_^2+^ photocatalyst is achieved via the reaction of Ru(bpy)_3_^+^ with atmospheric oxygen, resulting in the production of superoxide anions (O_2_^•−^) as byproducts. In the confined environment of the electrospray process, the highly oxidizing O_2_^•−^ species abstract hydrogen atoms from the amine radical cation to yield the expected dehydrogenated products (Fig. 1b)^38^.

We operated this nESI MS photoreaction platform in two modes: (i) real-time reaction screening in which both the laser source for catalyst excitation and the DC voltage for charged droplet generation were applied simultaneously. This mode of analysis allows the detection of short-lived reaction intermediates and consumes only 60 pmol (100 µM × 0.6 µL) of N-heterocyclic analyte and 3 pmol (5 µM × 0.6 µL) of Ru(bpy)_3_^2+^ catalyst per analysis; (ii) in a second mode of operation, the DC voltage is turned off while the laser source is applied for a specified excited-state reaction duration (example 2 min, 5 min reaction times) at the tip of the glass capillary. Subsequent application of DC voltage enables *in-situ* MS characterization of reaction products formed at the capillary tip. In both experiments, the high fluence of light directed at the minuscule sample volume (~0.6 μL) present at the glass tip permits accelerated photoreaction screening in a matter of seconds.

### Comparing reactivity of tetrahydroquinoline and tetrahydroisoquinoline

At the onset of the study, we sought to confirm reports demonstrating incomplete aerobic oxidation in THiQ compared to THQ^7,35,36,39,40^. Using Ru(bpy)_3_^2+^, photoredox oxidation of 1,2,3,4-tetrahydroquinoline (***1***, *MW* 133) proceeded smoothly to yield the expected quinoline dehydrogenated product (***3***, *m/z* 130) via the removal of four hydrogen atoms (Fig. 1d). The conversion rate was calculated to be 91% in just 2 min of laser exposure time. Reaction yields for these initial evaluations were based on MS data using the absolute intensities of the product of interest relative to total ion intensity for all products and unreacted reagents^38,41^. In the absence of visible light, however, no dehydrogenation products were observed; instead, protonated species of ***1*** were detected at *m/z* 134 (Fig. 1c) indicating the observed dehydrogenation process is due to photochemical activity of Ru*(bpy)_3_^2+^. Similar experiment was performed using 1,2,3,4-tetrahydroisoquinoline (***4***, *MW* 133) but in this case, the dihydroisoquinoline intermediate (***5***, via the removal of only two hydrogen atoms) was detected at *m/z* 132 (Fig. 1f), irrespective of visible light exposure time. These results are consistent with previous reports in which aerobic oxidative dehydrogenation of THiQ have always ended in the formation of the corresponding imine. The data also argues against a concerted dehydrogenation process by which the four hydrogen atoms may be removed. Instead, tautomerization of the initially formed C=N bond to a C=C bond (e.g., ***2i*** → ***2ii***, Fig. 1b) is necessary to enable the second dehydrogenation from the cyclic secondary amine.

### Reduced reactivity of THiQ revealed by theoretical calculations

**Figure 2:**
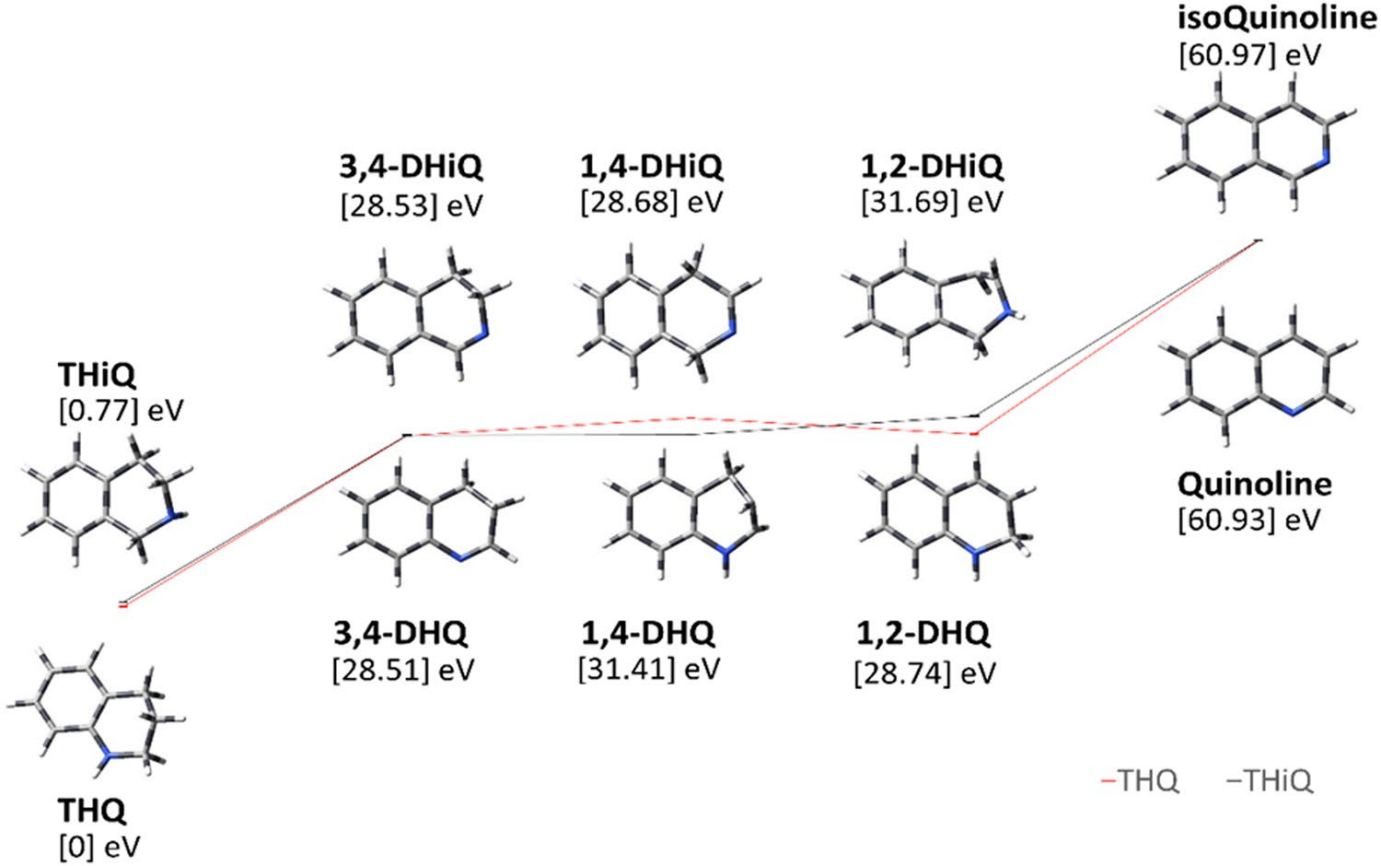
Schematic of relative energies of reactants (THQ, THiQ), intermediates (dihydro THQ/ThiQ) and products (quinoline, isoquinoline) compared to the energy of THQ. Red trace represents energy levels of quinolines while black trace represents isoquinoline.

DFT calculations indicated tautomerization in THQ and THiQ should occur at comparable rates to give intermediates that have comparable thermodynamic stabilities (Fig. 2). Ionization potentials for each intermediate were also calculated and determined to be comparable (Table S1). A close examination of the optimized structures for dihydroisoquinolonilne (DHiQ) intermediates (Fig. S1) revealed that all intermediates (***5***) have a double bond character at the second position (on N atom); the double bond character in ***5iii*** intermediate is formed through hyperconjugation^42^, which was confirmed by a shorter C-N bond distance of 1.37 Å (Table S2) as opposed to 1.43 Å for typical C-N single bonds (bond length for C=N bond is 1.38 Å). These computational findings led us to conclude that for effective aerobic dehydrogenation of THQ/THiQ, a cyclic secondary amine intermediate, with sp^3^ nitrogen, must be generated from the tautomerization process. To investigate this expectation, we proposed the use of N-substituents with electron donating abilities to reduce the C-N double bond character in ***5iii*** (1,2-DHiQ). DFT structural optimization of a *pi*-donating N-phenyl derivative of THiQ showed significantly increased C-N bond length from 1.37 Å in 1,2-DHiQ to 1.50 Å in phe-1,2-dihydroisoquinoline (phe-1,2-DHiQ; Fig. S1 and Table S2).

### Electrospray-based real-time reaction screening experiments confirm theoretical findings

**Figure 3:**
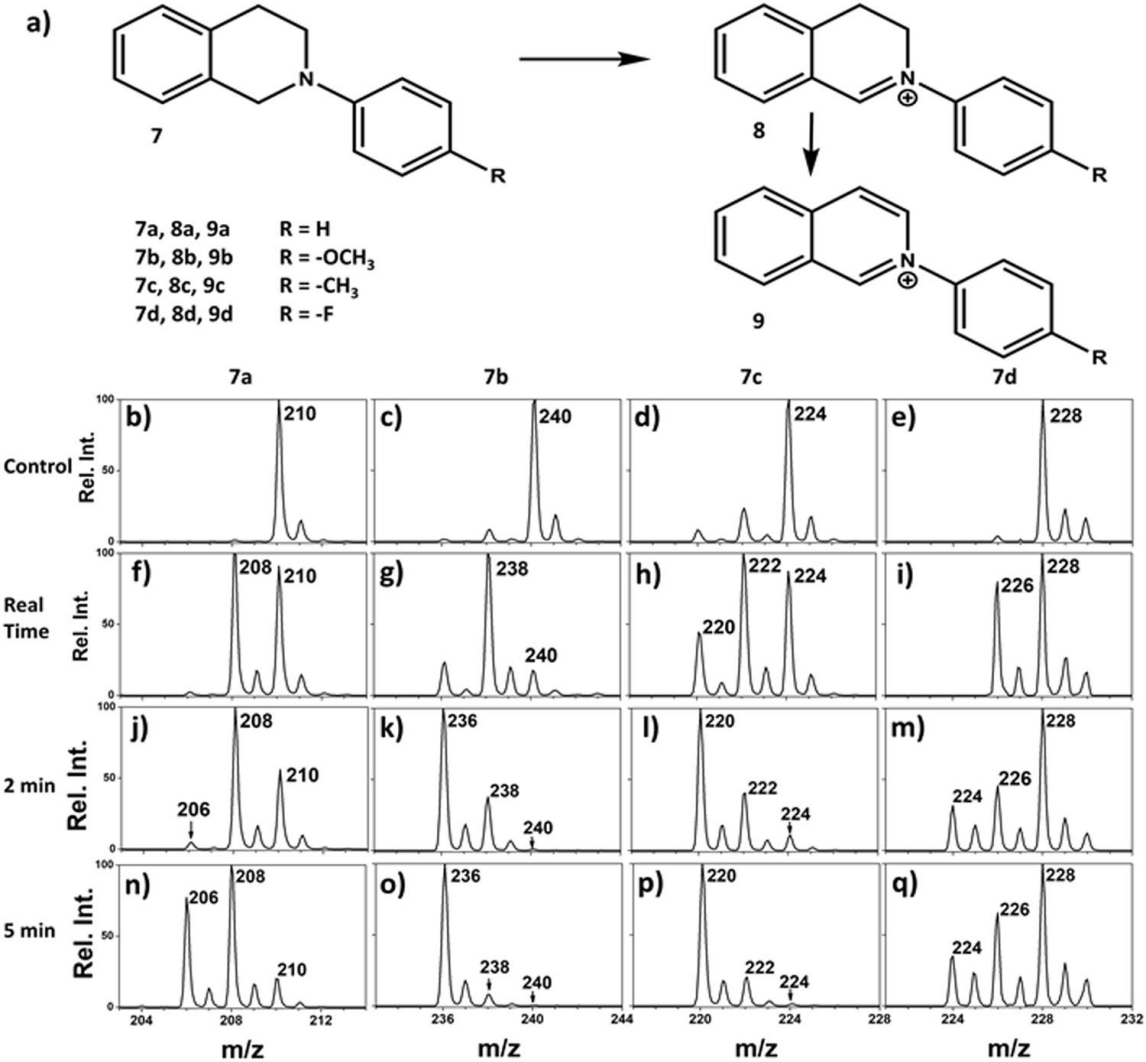
(**a**) Schematic representing dehydrogenation of N-substituted THiQ **7** to produce N-substituted dihydroisoquinoline, ***8***, which further oxidizes to give N-substituted isoquinolines, ***9*** as the main product. (**b–q**) Mass spectra showing analysis of dehydrogenation reaction mixture of ***7*** to ***9*** using the real-time photoreaction screening platform. The first row represents controls for each reactant, mass spectra for 100 µM ***7*** and 5 µM of Ru(bpy)_3_^2+^ in ACN without irradiation. The second row represents real time, mass spectra recorder after simultaneous application of DC spray voltage and laser irradiation. The third and fourth rows represent mass spectra recorded after continuously irradiation of the reaction mixture for 2 min and 5 min, respectively.

To confirm results from DFT calculations, a preliminary experiment was conducted on our nESI MS photoreaction screening platform using 2-phenyl-1,2,3,4-tetrahydro-isoquinoline, (***7***; 2-phe-THiQ, Fig. 3a). In the absence of light, the reaction mixture, which consisted of ***7a*** (100 µM; *MW* 209 Da) and Ru(bpy)_3_Cl_2_.6H_2_O (5 µM), produced only the protonated species of ***7a*** at *m/z* 210 during nESI MS analysis (Fig. 3b). In the presence of blue light, however, intermediate ***8a*** (yield 53.91%) was observed within seconds of laser exposure (real time, Fig. 3f), which corresponds to a 2 Da decrease in mass compared to the starting material, ***7a***. Continuous exposure of the blue coherent light to the capillary tip for 2 min not only increased the yield for the intermediate to 67.51%, but it also produced the expected product, 2-phenyl-isoquinoline, ***9a*** (yield 3.19%) at *m/z* 206 (Fig. 3j). Prolonged light exposure (5 min) increased the product yield from 3.19% to 31.55% (Fig. 3n). These results are very similar to those recorded for THQ where complete dehydrogenation (removal of 4 H atoms) is achieved without N-substitution (Fig. 1d). *To the best of the authors’ knowledge*, *this report represents the first record on complete aerobic oxidative dehydrogenation of any derivative of THiQ*. The use of visible light, atmospheric air, and off-the-shelf Ru(bpy)_3_^2+^ photocatalyst are added advantages.

### Comparing rate of dehydrogenation among *p*-phenyl substitution

Having established that the manipulation of electron density on N atom influences the reactivity of THiQ, we further investigate the effect of *p*-substituents (R = −F, −H, −CH_3_, −OCH_3_; increasing order of electron donation) on the rate of the dehydrogenation reaction. These N-substituted phenyl-1,2,3,4-THiQ derivatives [***7b*** (*MW* 239), ***7c*** (*MW* 223), and ***7d*** (*MW* 227)] were synthesized and tested on the nESI MS photoreaction screening platform. As expected, these *p*-substituted N-phenyl derivatives behaved similarly compared to 2-phe-THiQ (***7a;*** R = −H) where two consecutive dehydrogenation steps occurred under the current aerobic oxidation conditions. In the presence of light and photocatalyst Ru(bpy)_3_^2+^, all the N-phenyl derivatives produced the expected products (Fig. 3b–q), and yields increased for both intermediates and final products after increasing irradiation time. For example, 16.58% yield was calculated for ***7b*** (R = −OCH_3_) during real time analysis, which increased to 71.03% and 89.62% for 2 and 5 min of laser exposure times, respectively (Fig. 3c,g,k,o). Similar time-resolved analyses in yields for intermediates and product are summarized in Table S3 for all derivatives tested. The rates of dehydrogenation can be estimated based on the time-resolved yields. Based on optimized structure calculations, we expect reaction rates to increase with increasing electron donation ability. This expectation was found to be true by considering the total conversions (Table 1). The highest reaction rate was recorded for the *p*-methoxy N-phenyl derivative (***7b***), which has the highest electron donating ability, followed by *p*-methyl (***7c***) and *p*-fluoro (***7d***) N-phenyl derivatives, in decreasing order. A linear free energy relationship (Fig. S2) was generated by comparing the reaction rates of the *p*-substituted N-phenyl derivatives to the rate recorded for R = H in N-phenyl derivative (***7a***). The observed negative slope confirms a positive charge build up at the reaction center, and that the electron donating groups stabilize the resultant charge through resonance and induction effects^43^.

**Table 1:** Percentage yield calculated for various N-substituted derivatives after 2 minutes irradiation time.

N-Derivative	Conversion Rate (%)^#^					
	Intermediate		Main Product		Total Conversion	
	Average	SD	Average	SD	Average	SD
7a	67.5	2.9	3.19	1.2	70.7	2.8
7b	28.3	1.2	71.0	1.2	99.4	0.06
7c	26.7	2.5	66.6	2.9	93.3	0.7
7d	27.7	1.3	17.9	1.2	45.6	2.4

SD = standard deviation; average reported of 5 repetitive trials

^**#**^Like previous studies^38,41^, conversion rates were evaluated by the parameter of relative ion intensities, which compares the intensity of a specific ion (reactant or product) to the sum of intensities of product, reactant and intermediates derived from a reactant (see experimental section in supplemental information for details). This calculation is based on the assumption that products and starting materials have similar ionization/detection efficiencies in the MS analysis.

### Rational photocatalytic design based on N-methyl auxiliary

**Figure 4:**
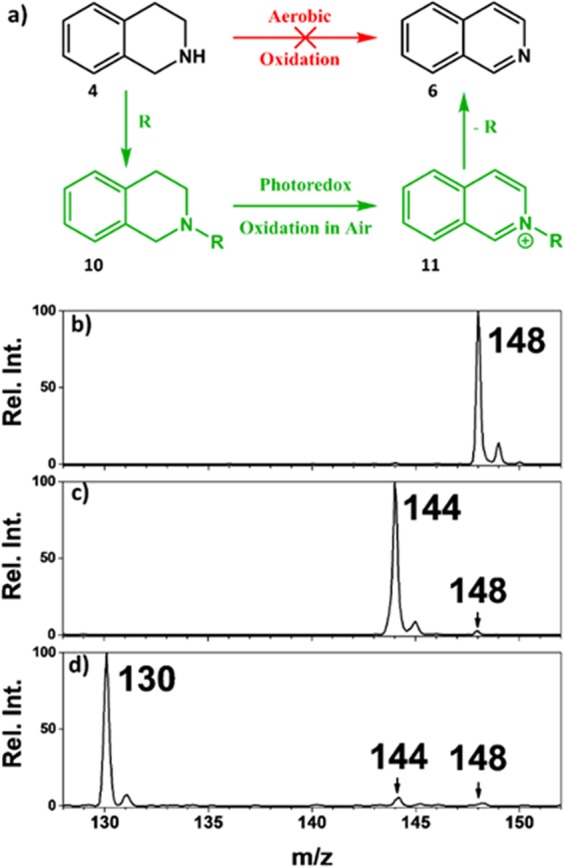
(**a**) Schematic illustrating the rational catalytic design (green pathway) proposed in this work, which represents three simple steps to synthesize isoquinolines **6** from **4**: step 1 – activation of THiQ via N-methyl auxiliary substitution, step 2 – aerobic oxidative dehydrogenation using Ru(bpy)_3_^2+^ photo catalyst, and step 3 – regeneration of isoquinolines via the removal of methyl auxiliary. Mass spectra showing (**b**) N-methyl substitution of **4** to produce species at *m/z* 148, (**c**) aerobic oxidative dehydrogenation of 10 (4 h in sunlight) to form **11** (*m/z* 144) via the removal of 4 H atoms, and (**d**) removal of methyl auxiliary to the expected final isoquinoline product at *m/z* 130.

Although N-substituted isoquinoline derivatives occur in many alkaloids and pharmacologically active compounds^44–46^ and are widely used as intermediates/reagents for various synthetic processes, the simple isoquinoline moiety is more common^47^. Therefore, a photocatalytic method based on the use of ambient air and capable of offering isoquinolines in an atom efficient manner will be most useful. For this, we rationally designed a novel synthetic strategy through the use of sigma (σ) donating methyl substituent and consisted of 3 simple steps illustrated in Fig. 4a: (i) first, we activated THiQ via N-substitution of the methyl auxiliary. DFT calculations showed that, this methyl auxiliary group reduces the sp^2^ character of C-N bond in the imine intermediate (methyl-1,2-DHiQ; Fig. S1 and Table S2); (ii) the second step involved visible light-promoted aerobic oxidative dehydrogenation of the activated N-methyl derivative in ambient air using the common Ru(bpy)_3_^2+^ photocatalyst; and (iii) lastly, we regenerated isoquinoline by demethylation using pyridinium hydrochloride. This demethylation step was chosen because it is selective^48^, cleaving only the N-methyl group. Although this approach is intended for scale-up preparative purposes, we first used the real-time nESI MS photoreaction screening platform to verify our new catalytic design. We used 2-methyl-THiQ for this initial screening test, and found a conversion rate of 78.6% in 5 min of light exposure (Fig. S3, Table S3). This rate enhancement is comparable to effect exerted by the *pi-*donating *p*-methyl N-phenyl THiQ derivative (***7c***; Table 1).

The photoredox reaction involving 2-methyl-THiQ was transferred from the droplet reaction system to bulk solution-phase conditions where the coherent laser source was replaced with sunlight. Exposure of 2-methyl-THiQ solution (5.0 mL: 2 mM, Fig. 4b) to photons from the sun resulted in 71.7% conversion rate within 4 h reaction time (Fig. 4c; also see NMR data in Fig. S5 after product purification). The resultant N-methyl isoquinoline was isolated, and subsequently subjected to 10 min demethylation with pyridinium hydrochloride reagent and the final pure isoquinoline product was produced with 76.9% conversion rate (Fig. 4d; see NMR data in Fig. S6). These results are significant because they represent the first report on auxiliary-based photocatalytic strategy for isoquinoline production enabled by the combination of theoretical calculations and real-time, rapid screening of experimental conditions. See Supplementary Information for step-by-step procedure for achieving large-scale synthesis of isoquinoline using the proposed method.

### Verification of reaction mechanism via kinetic studies

**Figure 5:**
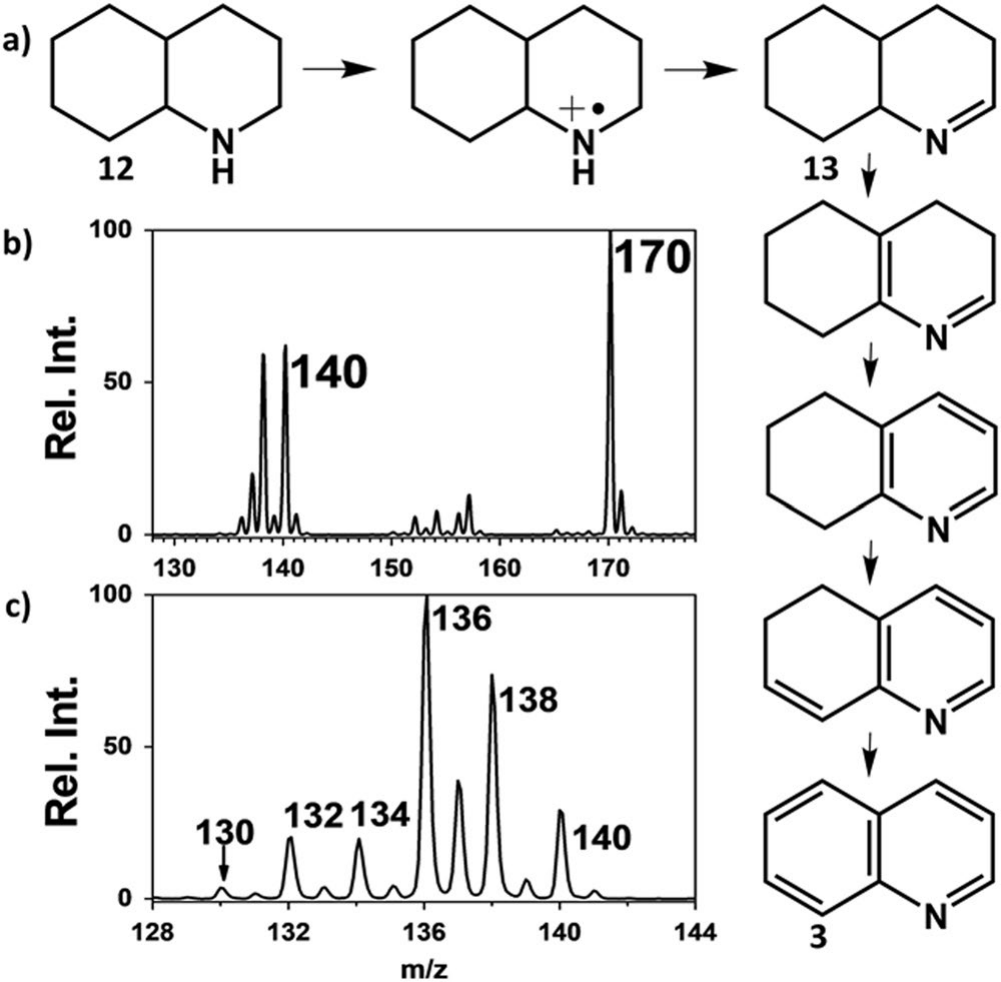
(**a**) Schematic illustration of dehydrogenation process resulting from the reaction of decahydroquinoline ***12*** with Ru(bpy)_3_^2+^ and leading to the formation of quinoline (***3***) through four successive tautomarization steps. Mass spectra recorded from a reaction mixture involving 100 µM of 12 and 5 µM Ru(bpy)_3_^2+^ in ACN bulk solution after: (**b**) 30 and (**c**) 120 minutes of laser exposure times.

To determine the validity of the proposed photocatalytic mechanism, we compared the rates of dehydrogenation for methyl derivative of THQ and THiQ. The rate of N-methyl THQ dehydrogenation was found to be 2X faster than that for N-methyl THiQ (Fig. S7, Table S3), which we attribute to the fact that only one tautomer in N-methyl THiQ can give rise to sp^3^ N, which is needed for dehydrogenation, compared to two active tautomers in N-methyl THQ. Finally, we subjected decahydroquinoline (***12***, *MW* 139) to our aerobic oxidative condition expecting to produce quinoline (***3***, *MW* 129) in five dehydrogenation steps, possible only if tautomerization allows for the formation of free sp^3^ nitrogen in a kinetically favorable manner (Fig. 5a). Irradiation of bulk solution to laser source (30 min) revealed a major reaction product at *m/z* 170 (Fig. 5b), which is identified by tandem MS and exact mass measurements to originate from molecular oxygen incorporation. (This product is probably formed via [2 + 2] cycloaddition involving ^1^O_2_ and the first dehydrogenation product from ***12***). Increasing the laser exposure time to 120 min yielded significant amounts of clean dehydrogenation products at *m/z* 136, 134, 132 and 130 (trace amounts) (Fig. 5c). This results clearly confirm that tautomerization leads to the movement of *π*-electrons through the fused ring, freeing the N atom for further dehydrogenation which yields a completely unsaturated product, quinoline, ***3***. In a separate set of experiments, ***12*** was replaced with structural isomer decahydroisoquinoline. As expected, dehydrogenation was terminated after two dehydrogenation steps (Fig. S8), again confirming that tautomerization is limited when the nitrogen atom is placed in the second position, without reinforcement from N-substitution.

## Conclusions

In summary, we have developed a novel photocatalytic platform where simple N-methyl substitution activates tetrahydroisoquinolines toward a more effective aerobic oxidative dehydrogenation. This visible light mediated transformation is accomplished using sun energy, atmospheric oxygen and ambient temperature. The mechanism of this new photoredox reaction was studied via theoretical calculations, which identified hyper-conjugation as the main factor limiting the complete aerobic oxidation of tetrahydroisoquinololines. This finding was promptly verified experimentally using a photoreaction screening platform that is based on nano-electrospray emitter fitted with portable laser source. This experimental setup enabled real-time, quantitative monitoring of reaction conditions using mass spectrometry. Because the confined environment of the electrospray-based reaction screening closely resembles that of condensed-phase reaction conditions, it was straightforward to transfer the optimized screening conditions to solution-phase isoquinoline synthesis, where 71.7% total yield could be produced in less than 4 h of reaction time. We expect this simple but effective photocatalytic platform to transform green large-scale production of isoquinolines, which form one of the most common commodity chemicals in pharmaceutical industry.

## Methods

### Mass spectrometry

A Velos Pro LTQ linear ion trap mass spectrometer (Thermo Scientific, San Jose, CA, USA) was used for online and off-line photoreaction studies, operated in the full mass spectrum mode and specific product ions produced by collision-induced dissociation (CID). MS parameters used were as follows: 200 °C capillary temperature, 3 microscans, and 60% Slens voltage. Spray voltage was 1.2 kV unless otherwise specified. Orbitrap™ mass spectrometer (Exactive™ Plus EMR, Thermo Scientific, San Jose, CA, USA) was used for high resolution electrospray ionization mass spectra. During experiments inlet capillary was maintained at 200 °C and Helium gas was employed as the collision gas. All the experiments were conducted in positive ion mode and 15–25% optimal normalized collision energy was used with CID. Data were acquired and processed using Xcaliber 2.2 (Thermo Scientific) software. The nano-electrospray ionization (nESI) capillary were pulled from borosilicate glass capillaries with filament (Sutter Instrument, USA) using a micropipette puller (Model P-97, Sutter Instrument Co., Novato. CA, USA). Laser pointer (450 nm, 5 mW) was purchased from Beam of Light Technologies, Inc.

### Computational calculations

For electronic structure calculations Density functional theory (DFT) was used on reactants, intermediates (tautomers), and products utilizing the Gaussian 09 suit of programs^49^. The Becke three parameter correlation functional combined with Lee, Yang and Parr exchange functional was employed (B3LYP)^50,51^. And 6–31 g* basis set was employed in optimization process of C, H, N & O. The compound structures were optimized in the gas phase to a minimum (confirmed by the absence of imaginary frequencies in vibrational analysis). GaussView 5.0 was employed in visualization of the molecules.

### Chemicals & reagents

Chemicals and solvents were used without further purification as they were of the highest commercial grade, unless otherwise stated. 1,2,3,4-Tetrahydroquinoline, 1,2,3,4-Tetrahydroisoquinoline, Tris(2,2-bipyridyl)dichlororuthenium(II)hexahydrate, (Ru(bpy)3Cl2.6H2O), Tris(dibenzylideneacetone)dipalladium(0) (Pd_2_(dba)_3_), 2,2′-Bis(diphenylphosphino)-1,1′-binaphthalene (BINAP), Bromobenzene, 4-Iodoanisole, 4-Iodotoluene, 4-Fluroiodobenzene, Methyliodide, Sodium tert-butoxide, Pyridine chloride, Ammonium hydroxide, Magnesium sulfate, Formic acid, triethylamine, Pottasium carbonate, (1Z,5Z)-cycloocta-1,5-diene and all the solvents (Acetonitrile, trideuteroacetonitrile, Toluene, Dichloromethane, Cyclohexane, Tetrahydrofuran, Ethyl acetate, Ethanol, Chloroform) were purchased from Sigma-Aldrich. Decahydroquinoline and Decahydroisoquinoline were purchased from Ark Pharm, Inc. All the N-derivatives of tetrahydroquinoline and tetrahydroisoquinoline were synthesized in-house under N_2_ environment (see supporting information for synthetic details).

## Supplementary information

Supplemental Information
