# Supplementary material for: N-Substituted Auxiliaries for Aerobic Dehydrogenation of Tetrahydro-isoquinoline: A Theory-Guided Photo-Catalytic Design
Source: Sci Rep. 2019 Aug 2;9:11280. doi: 10.1038/s41598-019-47735-y (PMC6677888; doi:10.1038/s41598-019-47735-y)
Supplement: Supplementary file 1 — Supplemental Information [file 41598_2019_47735_MOESM1_ESM.pdf]

Supporting Information

N-Substituted Auxiliaries for Aerobic Dehydrogenation of Tetrahydro-isoquinoline: A Theory-Guided Photo-Catalytic Design

Savithra Jayaraj[1] and Abraham K. Badu-Tawiah[1,*]

[1]Department of Chemistry and Biochemistry, The Ohio State University, Columbus OH 43210

*Correspondence to badu-tawiah.1@osu.edu

Supporting Information

Table of Content

Topic	Title of the Topic	Page
**Topic 1**	Experimental Section: Materials, Apparatus and Experiments	3
**Topic 2 (Figure S1)**	DFT calculation of thermodynamic stabilities of THQ, THiQ and their intermediates	5
**Topic 3 (Table S1)**	Calculation of ionization energies of THQ, THiQ and their intermediates	6
**Topic 4 (Figure S2 & Table S2)**	Comparison of bond character and distance between different dihydro-THQs, THiQs and N-derivatized THiQs	7
**Topic 5 (Table S3)**	Time resolved analysis for yields of THiQ and N-derivatized THiQs	8
**Topic 6 (Figure S3)**	Linear free energy correlation of the photoreaction	9
**Topic 7 (Figure S4)**	Real-time experimental verification of theoretical data on N-methyl THiQ oxidation	10
**Topic 8 (Figure S5)**	NMR characterization of product ensuing from N-methyl substitution of THiQ	11
**Topic 9 (Figure S6)**	NMR characterization and confirmation of product from photoredox oxidation of N-methyl THiQ	12
**Topic 10 (Figure S7)**	NMR characterization and confirmation of product obtained after demethylation of N-methyl THiQ	13
**Topic 11 (Figure S8)**	Comparison of reaction rate for photoredox oxidation of N-methyl derivatives of THQ and THiQ	14
**Topic 12 (Figure S9)**	Comparison of photoredox reaction between decahydroquinoline and decahydroisoquinoline	15
**Topic 13**	References	16

Topic 1: Experimental Section

Materials

Chemicals and solvents were used without further purification as they were of the highest commercial grade, unless otherwise stated. 1,2,3,4-Tetrahydroquinoline, 1,2,3,4-Tetrahydroisoquinoline, Tris(2,2-bipyridyl)dichlororuthenium(II)hexahydrate, (Ru(bpy)3Cl2.6H2O), Tris(dibenzylideneacetone)dipalladium(0) ($Pd_2(dba)_3$), 2,2'-Bis(diphenylphosphino)-1,1'-binaphthalene (BINAP), Bromobenzene, 4-Iodoanisole, 4-Iodotoluene, 4-Fluroiodobenzene, Methyliodide, Sodium tert-butoxide, Pyridine chloride, Ammonium hydroxide, Magnesium sulfate, Formic acid, triethylamine, Pottasium carbonate, (1Z,5Z)-cycloocta-1,5-diene and all the solvents (Acetonitrile, trideuteroacetonitrile, Toluene, Dichloromethane, Cyclohexane, Tetrahydrofuran, Ethyl acetate, Ethanol, Chloroform) were purchased from Sigma-Aldrich. Decahydroquinoline and Decahydroisoquinoline were purchased from Ark Pharm, Inc. All the N-derivatives of tetrahydroquinoline and tetrahydroisoquinoline were synthesized in-house under N_2 environment.

Apparatus

Laser pointer (450 nm, 5 mW) was purchased from Beam of Light Technologies, Inc.

The nano-electrospray ionization (nESI) capillary were pulled from borosilicate glass capillaries with filament (Sutter Instrument, USA) using a micropipette puller (Model P-97, Sutter Instrument Co., Novato. CA, USA).

A Velos Pro ion trap mass spectrometer (Thermo Scientific, San Jose, CA, USA) was used for online and off-line photoreaction studies, operated in the full mass spectrum mode and specific product ions produced by collision-induced dissociation (CID).

Orbitrap™ mass spectrometer (Exactive™ Plus EMR, Thermo Scientific, San Jose, CA, USA) was used for high resolution electrospray ionization mass spectra. During experiments inlet capillary was maintained at 200 ^{0}C and Helium gas was employed as the collision gas. All the experiments were conducted in positive ion mode and 15-25% optimal normalized collision energy was used with CID. Data were acquired and processed using Xcaliber 2.2 (Thermo Scientific) software.

Bruker DMX- 400 spectrometer was used for ^{1}H NMR studies with CD_3CN as the solvent.

Reactions in Photo-reaction Screening Platform

During online screening experiments 60 pmol (100 µM × 0.6 µL) of N-heterocyclic analyte and 3 pmol (5 µM × 0.6 µL) of $Ru(bpy)_3^{2+}$ catalyst was consumed per analysis.

Experiments were performed on in-house developed picomole-scale photoreaction screening platform, (Figure 2), which involves the coupling of portable laser source (wavelength 450 nm, power 5 mW) with nano-electrospray ionization (nESI) emitter. Application of 1.2 kV DC voltage to reaction mixture produced charged droplets, containing the reactants, which were directly transported to mass spectrometer (MS). In-situ exposure of these charged droplets to the blue coherent laser light (kept ~0.8 cm distance from the transparent glass capillary nano-tube) initiated photochemical reaction under ambient conditions, and products were promptly characterized by MS in real time. For prolong reaction (example 2 min, 5 min reaction times), the DC voltage was turned off while the blue coherent light was continuously directed at the transparent glass nano-tube. The observed reaction yields were calculated using the following equation[1]:

$$Observed\ Yield = \frac{I\ product}{(I\ reagent + \Sigma I\ all\ products)} X\ 100\%$$

Where I = Absolute intensities; I product = Product of interest; ΣI all products = all other observed products.

This calculation assumes that products and starting materials have similar ionization. Similar approach has been used in previous studies[1,2].

Different N-derivatives of THiQ and THQ were tested using this platform under ambient conditions.

Summary of procedure for large-scale solution-phase synthesis of isoquinoline

The following three steps were used for large scale solution-phase synthesis of isoquinoline: first, we synthesized N-methyl-1,2,3,4-tetrahydroisoquinoline according to method developed by Matthijs J. van Haren et al (see Supplemental for details). Isolate yield for this N-methylation step was 21% using NMR spectroscopy using (1Z, 5Z)-cycloocta-1,5-diene as an internal standard. Second, the synthesized N-methyl-1,2,3,4-tetrahydroisoquinoline (5.0 mL; 2 mM) was exposed to sunlight for 4 h using a 5 mol% catalyst $[Ru(bpy)_3]^{2+}$ loading. Product yield for this photoredox reaction was 71.7%, which was monitored with NMR spectroscopy using (1Z, 5Z)-cycloocta-1,5-diene as an internal standard. Last, purified N-methyl isoquinoline was subjected to 10 min demethylation step. Here, 1.0 g of anhydrous boiling pyridinium chloride was added to the 0.1 mmol 2-methyl-1,2,3,4-tetrahydroisoquinolinium and the resulting mixture was refluxed for 10 minutes under N_2. The resulting hot solution was then transferred into a mixture of ice cold concentrated ammonia. The aqueous layer was then extracted with ethyl acetate followed by drying over magnesium sulfate. The resulting solution

was evaporated to remove solvent and purified by column chromatography on silica gel using cyclohexane/ethyl acetate as eluent to yield isoquinoline as a light-yellow liquid. Isolate yield of the final isoquinoline product was 76.9%, also determined using NMR spectroscopy.

Synthesis of Derivatives

2-Aryl-1,2,3,4-tetrahydroisoquinoline

All 2-aryl-1,2,3,4-tetrahydroisoquinolines were synthesized by a modified method of Jia-Xing Jiang et al[3].

2-Phenyl-1,2,3,4-tetrahydroisoquinoline: $Pd_2(dba)_3$ (57 mg, 0.1 mmol), BINAP (125 mg, 0.2 mmol) were added into an oven dried schlenk flask (50 mL) and degassed with N_2. To this 10 mL of freshly distilled, degassed Toluene was introduced through a syringe. This was allowed to stir in 110 ^{0}C for 45 min before cooling it down to room temperature. To the same flask 1,2,3,4-tetrahydroisoquinoline (0.67 g, 5 mmol), Bromobenzene (468 mg, 3 mmol), and NaOtBu (480 mg, 5 mmol) were added and the mixture was then degassed with N_2 and refluxed under N_2 for 8hrs. The mixture was allowed to cool down before filter through celite. The celite was washed with DCM (5 x 5 mL). The solvent was removed by rotatory evaporation and the crude product was purified by column chromatography on silica gel using cyclohexane/ethyl ether as eluent. The product was confirmed through Mass Spectrometry.

1-Methyl-1,2,3,4-tetrahydroquinoline

N-methyl-1,2,3,4-tetrahydroquinoline was synthesized according to the method reported in Xue Jiang et al[4].

4 mmol of 1,2,3,4-tetrahydroquinoline was added to a solution containing 80 mmol of HCOOH and 80 mmol of triethylamine and degassed using N_2 for 15 minutes. The reaction mixture was stirred overnight at 150^{0}C under N_2 environment. The resulting solution was allowed to come to room temperature and basified with NaOH solution. Then the mixture was extracted with CH_2Cl_2 and purified by column chromatography on silica gel using cyclohexane/ethylacetate as eluent to yield 1-methyl-1,2,3,4-tetrahydroquinoline as a yellow liquid.

2-Methyl-1,2,3,4-tetrahydroisoquinoline

N-methyl-1,2,3,4-tetrahydroisoquinoline was synthesized according to method by Matthijs J. van Haren et al[5].

4 mmol of 1,2,3,4-tetrahydroisoquinoline was dissolved in 20.0 mL ethanol and 6 mmol of potassium carbonate was added followed by 8 mmol methyl iodide. The mixture was allowed to stir overnight at room temperature. Then solvent was removed, re-dissolved in 25.0 mL of water and the product was extracted with $CHCl_3$ (3 × 25.0 mL). The crude product was purified by column chromatography on silica gel using cyclohexane/ethyl acetate as eluent to yield 2-methyl-1,2,3,4-tetrahydroisoquinoline as a dark yellow liquid.

Demethylation of 2-Methyl-1,2,3,4-tetrahydroisoquinoline to produce isoquinoline

Demethylation reaction was done according to method by Anthony Ruiz et al[6].

1.0 g of anhydrous boiling pyridinium chloride was added to the 0.1 mmol 2-methyl-1,2,3,4-tetrahydroisoquinolinium compound and the resulting mixture was refluxed for 10 minutes under N_2. The resulting hot solution was then transferred into a mixture of ice cold concentrated ammonia. The aqueous layer was then extracted with ethyl acetate followed by drying over magnesium sulfate. The resulting solution was evaporated to remove solvent and purified by column chromatography on silica gel using cyclohexane/ethyl acetate as eluent to yield isoquinoline as a light-yellow liquid.

Topic 2: Thermodynamic stabilities for 1,2,3,4-tetrahydroquinoline (THQ)/1,2,3,4-tetrahydroisoquinoline (THiQ), dihydro-intermediates of THQ/THiQ and products were calculated relative to the stability of THQ. These DFT calculations indicates tautomerizaation in THQ and THiQ should occur at comparable rates to give intermediates and products quinoline/isoquinolines as they have comparable stabilities. For example, an energy barrier of 3.16 eV was calculated to exist between the most stable intermediate (3,4-DHiQ) and the least stable 1,2-DHiQ intermediate for THiQ*;* this is similar to an energy barrier of 2.90 eV (between 3,4-DHQ and 1,4-DHQ) for THQ. These results suggest tautomerization is feasible in THiQ.

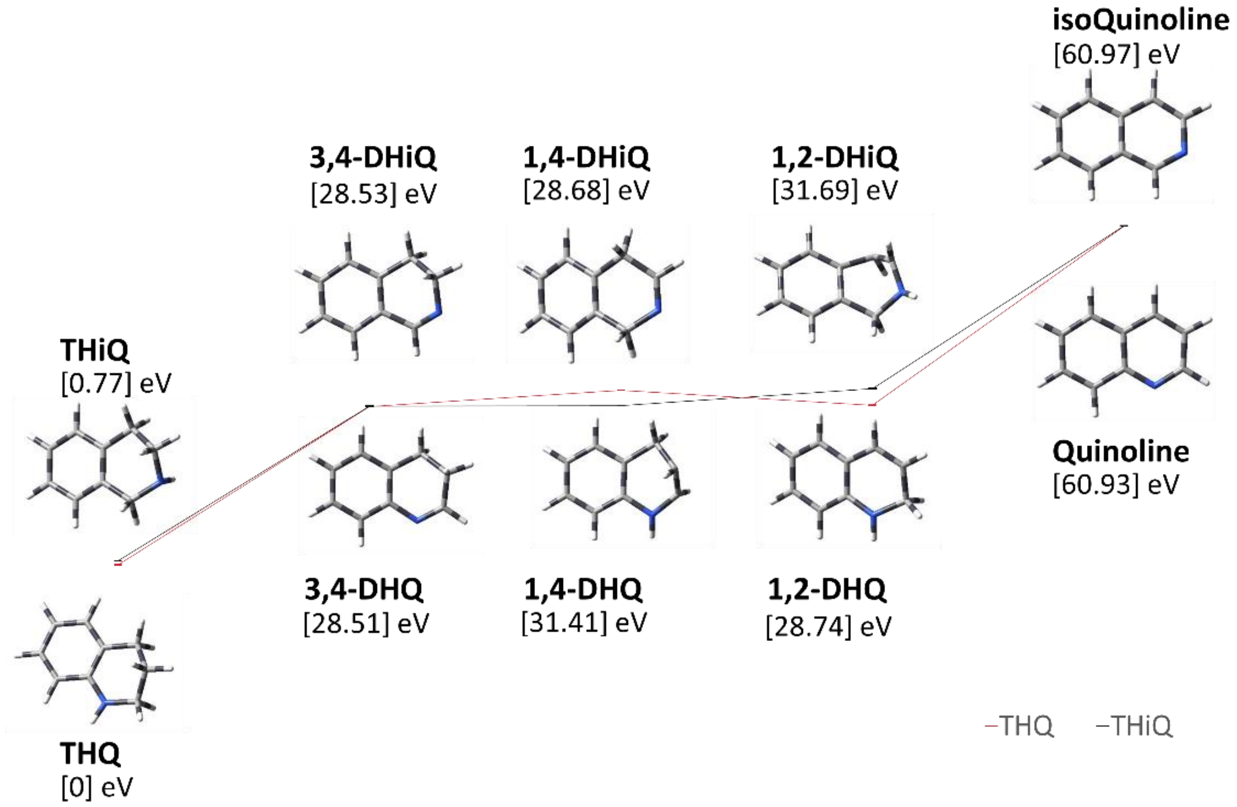

Figure S1: Relative energies of reactants (THQ, THiQ), intermediates (dihydro THQ/THiQ) and products (quinoline, isoquinolines) compared to the energy of THQ. Red trace represents energy levels of quinolines while black trace represents isoquinolines.

Topic 3: Ionization energies for 1,2,3,4-tetrahydroquinoline (THQ), 1,2,3,4-tetrahydroisoquinoline (THiQ) and their dihydro (DHQ/DHiQ) intermediates were calculated to compare energies to find the incomplete dehydrogenation of THiQ is due to the ionization potentials. However, we found that there is no significant difference in ionization energies of THQs and THiQs.

Table S1: Calculated ionization energies for model compounds

Compound	Ionization Energy (eV)
THQ	6.7*
3,4-DHQ	7.8
1,4-DHQ	3.8
1,2-DHQ	6.6
THiQ	7.6#
3.4-DHiQ	8.0
1,4-DHiQ	8.1
1,2-DHiQ	3.4

Experimental (photo electron spectroscopy) energy for *THQ = 7.0 eV[7], for #THiQ = 8.5 eV[8]

Note: Ionization potentials were calculated for the reaction shown below;

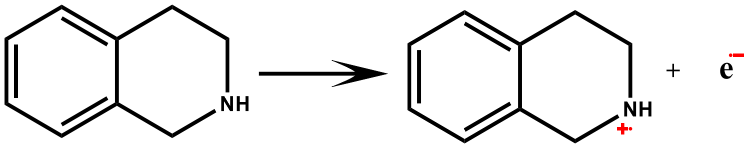

Topic 4: Bond distance between carbon and nitrogen of 1,2-dihydro intermediates were calculated to compare the bond character (single vs double). Only 1,2-dihydroisoquinoline (1,2-DHiQ) intermediate shows a double bond character. This double bond character can be attributed to the extended conjugation (hyperconjugation) which prevents further dehydrogenation of the molecule. When considering the derivates (methyl or phenyl) of 1,2-DHiQ, the hyperconjugation is limited to reinforce single bond character (sp^3 nitrogen) and complete dehydrogenation.

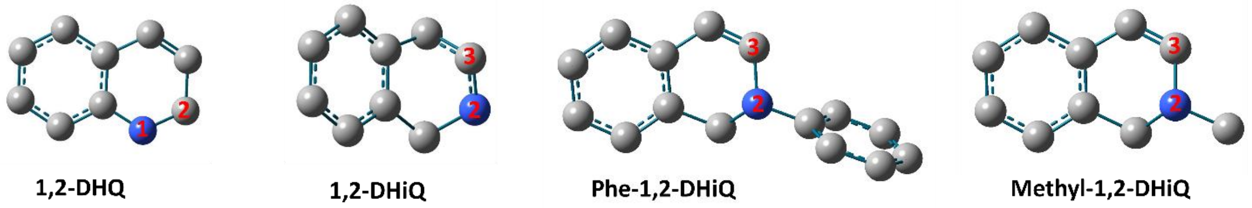

Figure S2: Geometry optimized structures for 1,2-dihydro intermediates of quinoline (1,2-DHQ), isoquinolines (1,2-DHiQ), phenyl substituted isoquinolines (Phe-1,2-DHiQ) and methyl substituted isoquinolines (Methyl-1,2-DHiQ). For 1,2-DHiQ, the double bond at position 3 and 4 extends to the nitrogen and introduces sp^2 character at position 2 that hinders dehydrogenation.

Table S2: Calculated bond distances for model compounds

	C-N bond distance (Å)	Bond Character
**1,2-DHQ (position 1 -2)**	1.46151	Single
**1,2-DHiQ (position 2 -3)**	1.36821	Double
**Methyl-1,2-DHiQ (position 2-3)**	1.49226	Single
**Phe-1,2-DHiQ (position 2 -3)**	1.49739	Single

Typical carbon nitrogen single bond distance (C-N) is 1.43 Å and double bond distance (C=N) is 1.38 Å.

Note: 1,2-DHQ, 1,2-DHiQ and their derivatives position labeled as follows. Where R = H, CH_3 or phenyl

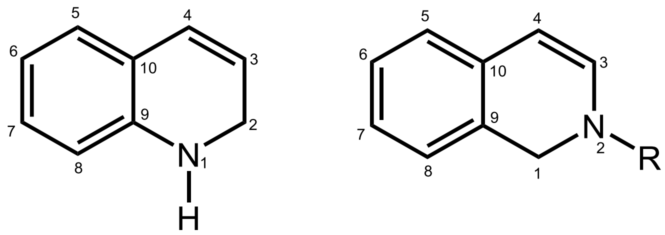

Topic 5: Time resolved analysis for yields were calculated for derivatives of THiQ and THQ from absolute intensities of Mass spectrum data for dehydrogenation by photoreaction screening platform. The total product yield increase with the increase of exposure of light. When comparing N-derivatives of THiQ, we can see that increase of donation ability by the R group in the N-derivatized THiQ increases the total product yield (7d < 7a < 7c < 7b). The N-methyl-THQ (14) is comparable to the 2-methoxyphenyl-THiQ(7b) in yields. Also, when comparing the N-methyl derivatives of THQ (14) and THiQ (16), we can see that the total yield of THQ derivative is higher than that of THiQ at each time constant (16 < 14). From these data, we can generate a reaction rate (increasing) order for the derivatives: 7d < 16 < 7a < 7c < 14 ≈ 7b

Table S3: Percentage yield calculated for N-derivatives of THiQ and THQ for real-time light exposure, 2 minutes and 5 minutes irradiation

Reactant	Real time						2 min						5 min					
	Intermediate		Main Product		Total		Intermediate		Main Product		Total		Intermediate		Main Product		Total Conversion	
	Yield%	SD	Yield%	SD	Yield%	SD	Yield%	SD	Yield%	SD	Yield%	SD	Yield%	SD	Yield%	SD	Yield%	SD
7a	53.91	2.52	1.23	0.05	55.14	2.49	67.51	2.90	3.19	1.22	70.70	2.80	50.86	7.22	31.55	13.00	82.41	10.24
7b	70.19	3.39	16.58	2.64	86.77	2.45	28.34	1.22	71.03	1.18	99.37	0.06	9.96	3.45	89.62	3.55	99.58	0.11
7c	38.85	2.53	19.81	1.39	58.66	2.14	26.70	2.47	66.56	2.89	93.26	0.68	23.80	7.84	74.69	8.07	98.49	0.24
7d	32.40	1.04	0.09	0.04	32.49	1.04	27.72	1.32	17.85	1.21	45.57	2.38	34.32	3.34	19.25	1.15	53.57	3.51
14*	5.00	2.92	0.05	0.03	5.05	2.94	42.11	13.23	36.33	13.31	78.44	0.09	24.25	8.30	54.36	8.39	78.61	0.18
16	5.46	0.38	51.59	1.16	57.05	1.16	7.53	0.73	82.67	4.37	90.20	4.71	9.24	5.05	90.37	5.13	99.61	0.09

* Control subtracted

Note: All the data reported are for 5 repetitive trials.

Yields were calculated using following equation;

$$Observed\ Yield = \frac{I\ product}{(I\ reagent + \Sigma I\ all\ products)} X\ 100\%$$

Where I = Absolute intensities; I product = Product of interest; ΣI all products = all other observed products.

Note: Detailed mass spectra for entry **7a, 7b, 7c** & **7d** can be found in main text **Figure 2** and entry **14** & **16** can be found in **Figure S4** and **S8.**

Topic 6: Linear free energy correlation was produced for the reaction by using different substituent groups at the para position of the N-phenyl substituent. Reaction rate was accelerated due to the presence of electron donating group; the highest reaction rate was recorded for the *p*-methoxy N-phenyl derivative (*7b*), which has the highest electron donating ability, and lowest for *p*-fluro (*7d*) N-phenyl derivatives. A negative slope was observed confirming a positive charge build up at the reaction center, and that the electron donating groups stabilize the charge built up.

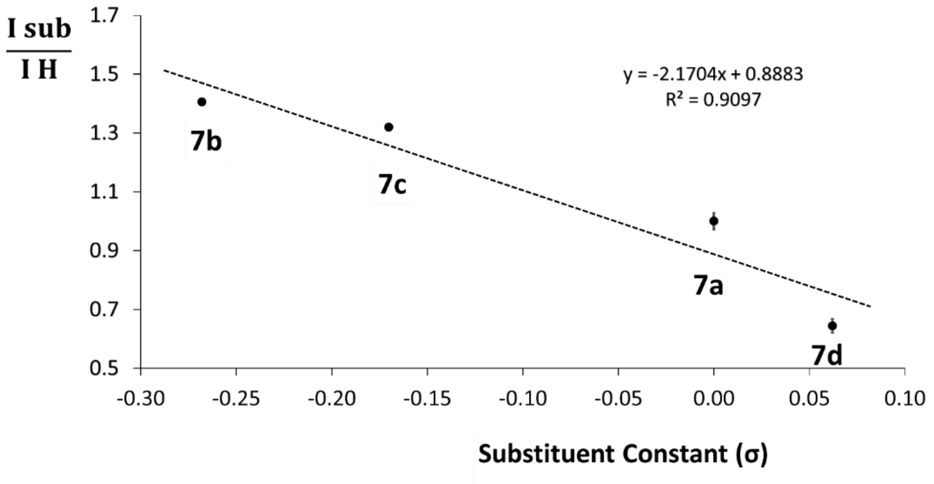

Figure S3: Reaction rate of each substituent was compared with that of the phenyl derivative, where $\frac{I\ sub}{I\ H}$ is plotted against the substituent constant, σ: I_{sub} = product ion intensity of the substituted derivative and I_H = product ion intensity of the unsubstituted phenyl derivative.

7a = 2-phenyl-1,2,3,4-tetrahydroisoquinoline

7b = 2-(p-methoxyphenyl)-1,2,3,4-tetrahydroisoquinoline

7c = 2-(p-methylphenyl)-1,2,3,4-tetrahydroisoquinoline

7d = 2-(p-flurophenyl)-1,2,3,4-tetrahydroisoquinoline

Topic 7: Verification of visible light promoted aerobic oxidative dehydrogenation using the N-methyl derivatized 1,2,3,4-tetrahydroisoquinoline (N-methylTHiQ, 14) - Mass spectra for dehydrogenation by photoreaction screening platform. This shows that dehydrogenation reaction proceeds to completion to generate N-methylisoquinolium, 15 with simple methylation on the nitrogen atom with comparative rates.

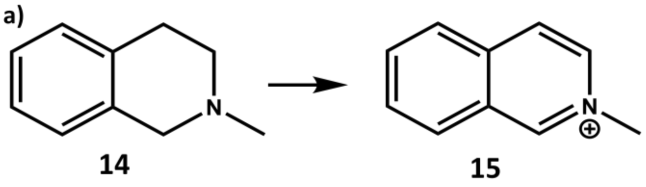

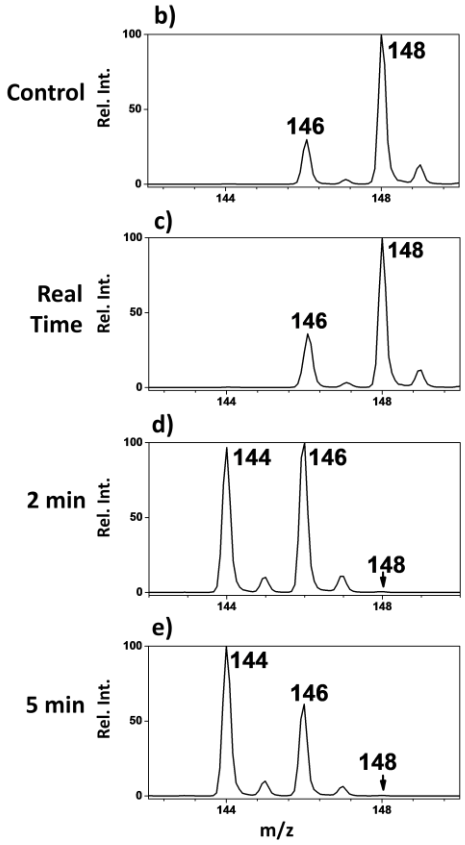

Figure S4: a) Schematic representing N-methyl-THiQ **14** dehydrogenation produce N-methylisoquinolium, **15** as the product; Mass spectra showing analysis of dehydrogenation of **14** into **15** using RPSP. The first row represent control, MS spectra for 100µM **14** and 5µM of $Ru(bpy)_3^{2+}$ in ACN without irradiation. The second row represents real time, MS spectra recorder after simultaneous application of DC spray voltage and laser irradiation. The third and fourth rows represent MS spectra recorded after continuously irradiating the reaction mixture for 2min and 5min, respectively.

Topic 8: Product confirmation and yield calculation for step 1: activation of THiQ via N-substitution of the methyl auxiliary for synthesis of isoquinoline from THiQ. Product yield was calculated to be 21.0% using (1Z, 5Z)-cycloocta-1,5-diene as an internal standard.

$$\text{Molar ratio} = \frac{\text{intensity of sample/no of H corresponding to sample peak}}{\text{intensity of standard/no of H corresponding to standard peak}} = \frac{1.05/2}{4.00/4} = 0.525$$

Molarity of sample = molarity of Standard × molar ratio = 20.0 Mm × 0.525 = 10.5 mM

$$\text{Percentage yield} = \frac{\text{experimental yield} \times 100\ \%}{\text{theoratical yield}} = \frac{10.5\ mM \times 100\%}{20\ mM} = 21.0\%$$

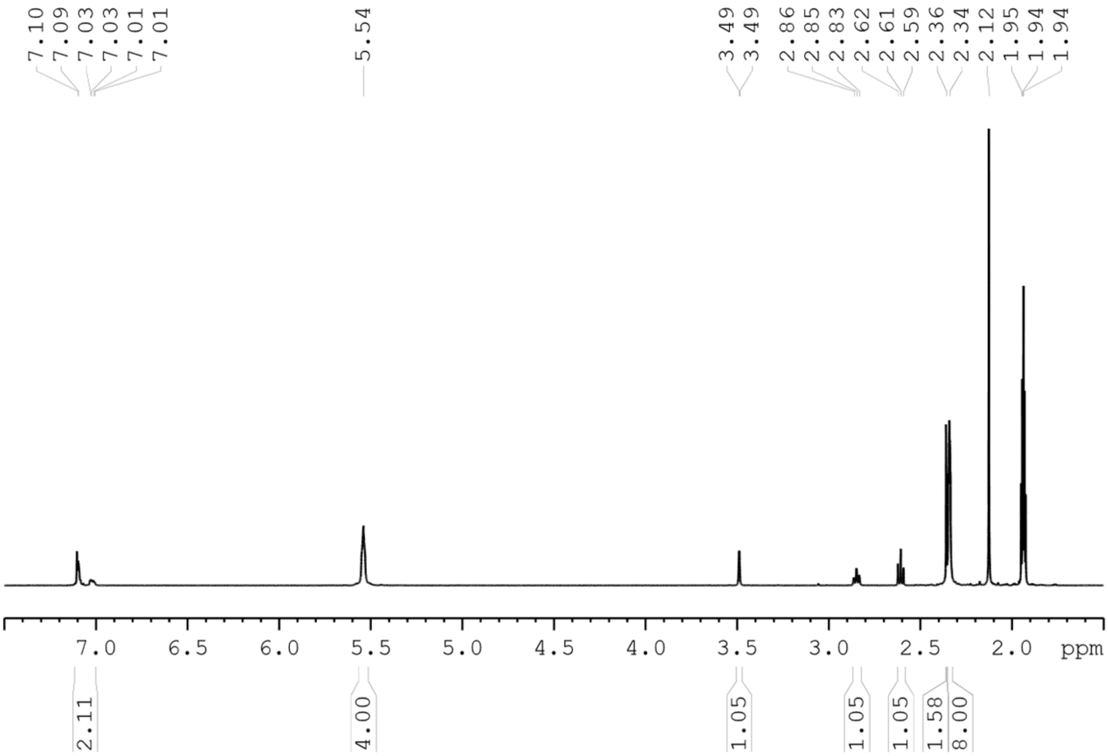

Figure S5: ^{1}H NMR of purified product of step 1: Synthesis of 2-methyl-1,2,3,4-tetrahydroisoquinoline (2-methyl-THiQ) and internal standard of (1Z, 5Z)-cycloocta-1,5-diene (CD_3CN, 400MHz, 300.2 K); product 2-methyl-THiQ was synthesized in bulk reaction and purified by column chromatography and the solvent was removed under vacuum and re-dissolved in 1.0 mL CD_3CN to which internal standard was directly added to calculate the yield as 21.0%. ^{1}H NMR of the (1Z, 5Z)-cycloocta-1,5-diene: δ 5.54 (s, 4H), 2.34 (s, 8H).

Topic 9: Product confirmation and yield calculation for step 2: visible light-promoted aerobic oxidative dehydrogenation of activated N-methyl-THiQ in ambient air using $Ru(bpy)_3^{2+}$ photocatalyst for synthesis of isoquinoline from THiQ. Product yield was calculated to be 71.7% using (1Z, 5Z)-cycloocta-1,5-diene as an internal standard. (with exposure of reaction mixture to sunlight for 4 hrs)

$$\text{Molar ratio} = \frac{\text{intensity of sample/no of H corresponding to sample peak}}{\text{intensity of standard/no of H corresponding to standard peak}} = \frac{1.075/3}{4.000/4} = 0.3583$$

Molarity of sample = molarity of Standard × molar ratio = 20.0 Mm × 0.3583 = 7.17 mM

$$\text{Percentage yield} = \frac{\text{experimental yield} \times 100\ \%}{\text{theoratical yield}} = \frac{7.17\ mM \times 100\%}{10\ mM} = 71.7\%$$

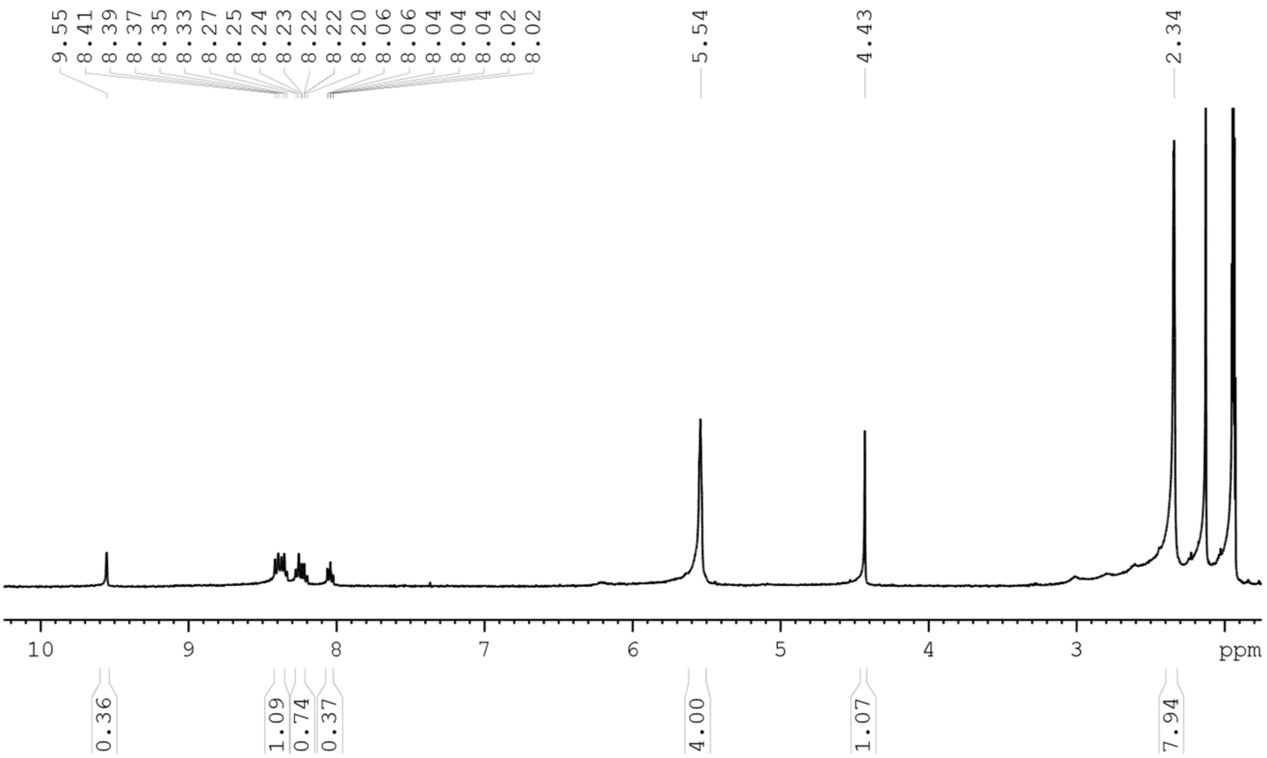

Figure S6: ^{1}H NMR of purified product of step 2: Dehydrogenation of 2-methyl-1,2,3,4-tetrahydroisoquinoline (2-methyl-THiQ) and internal standard of (1Z, 5Z)-cycloocta-1,5-diene (CD_3CN, 400MHz, 300.2 K); product 2-methylisoquinolinum ions were synthesized in bulk using sunlight as the light source, purified by column chromatography, the solvent was removed under vacuum and re-dissolved in 1.0 mL CD_3CN to which internal standard was directly added to calculate the yield as 71.7%. ^{1}H NMR of the (1Z, 5Z)-cycloocta-1,5-diene: δ 5.54 (s, 4H), 2.34 (s, 8H).

Topic 10: Product confirmation and yield calculation for step 3: regeneration of isoquinolines by demethylation using pyridinium hydrochloride. Product yield was calculated to be 76.9% using (1Z, 5Z)-cycloocta-1,5-diene as an internal standard.

Molar ratio = $\frac{\text{intensity of sample/no of H corresponding to sample peak}}{\text{intensity of standard/no of H corresponding to standard peak}} = \frac{1.066/1}{4.000/4} = 1.066$

Molarity of sample = molarity of Standard × molar ratio = 20.0 Mm × 1.066 = 21.3 mM

Mass of sample from NMR data = 2.75 mg

Total product measured after purification = 10.82 mg

Mass used for NMR reaction = 3.00 mg

Percentage yield = $\frac{\text{experimental yield} \times 100\ \%}{\text{theoratical yield}} = \frac{9.932\ mg \times 100\%}{12.916\ mg} = 76.9\%$

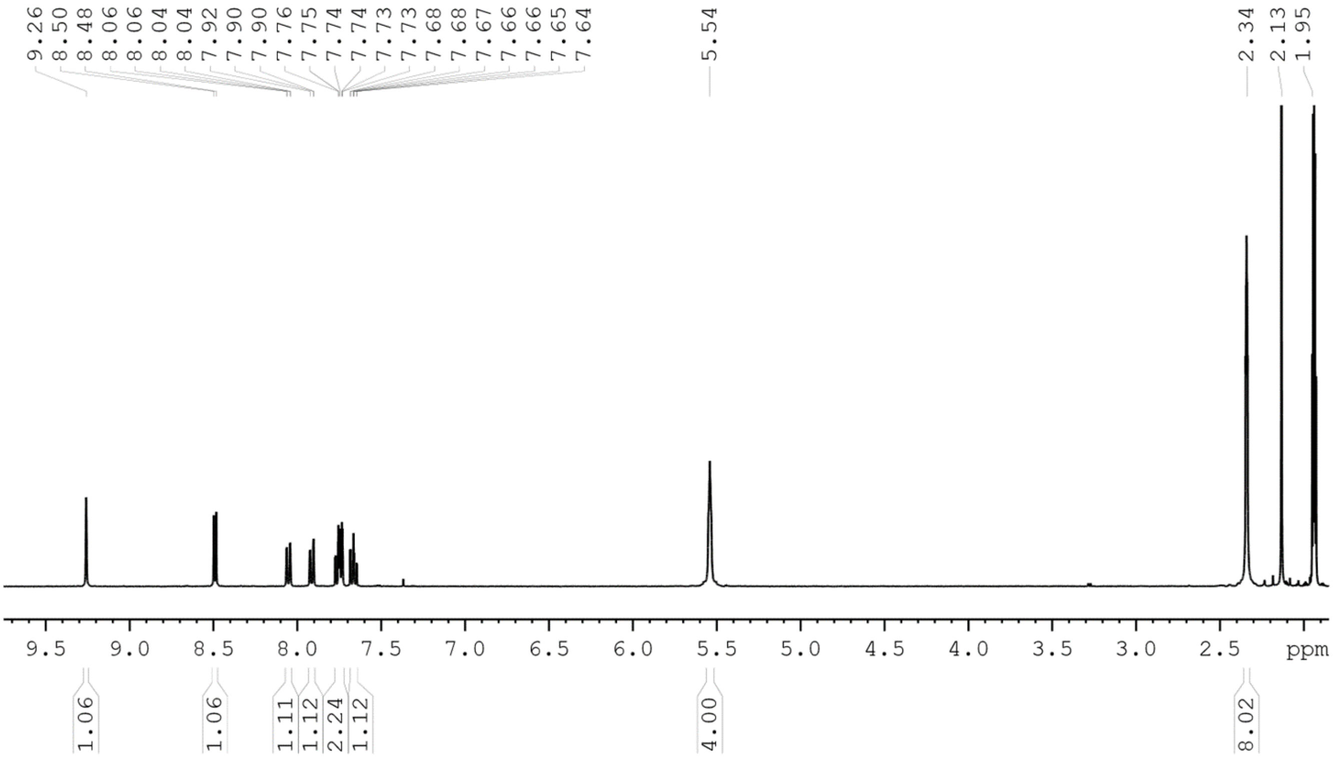

Figure S7: ^{1}H NMR of purified product of step 3: Regeneration of isoquinolines and internal standard of (1Z, 5Z)-cycloocta-1,5-diene (CD_3CN, 400MHz, 300.2 K); product isoquinolines was regenerated by demethylation with Py:HCl, purified by column chromatography, the solvent was removed under vacuum and re-dissolved in 1.0 mL CD_3CN to which internal standard was directly added to calculate the yield as 76.9%. ^{1}H NMR of the (1Z, 5Z)-cycloocta-1,5-diene: δ 5.54 (s, 4H), 2.34 (s, 8H).

Topic 11: Reaction rate comparison of 1,2,3,4-tetrahydroquinoline (THQ) and 1,2,3,4-tetrahydroisoquinoline (THiQ) using their methyl derivative - Mass spectra for dehydrogenation by photoreaction screening platform. Comparison of 14 and 16 shows that 16 reacts much faster and produces higher yield compared to 17. This can be attributed to the fact that both N-methyl-1,2-dihydroquinoline and N-methyl-1,4-dihydroquinoline tautomers can proceed to the next dehydrogenation in 16, while only the 1,2-dihydroisoquinoline can proceed to give the second dehydrogenation to produce N-methylquinolinum (17) and N-methylisoquinolinum (15) respectively as discussed in Topic 2 where the intermediates generate sp^3 nitrogen.

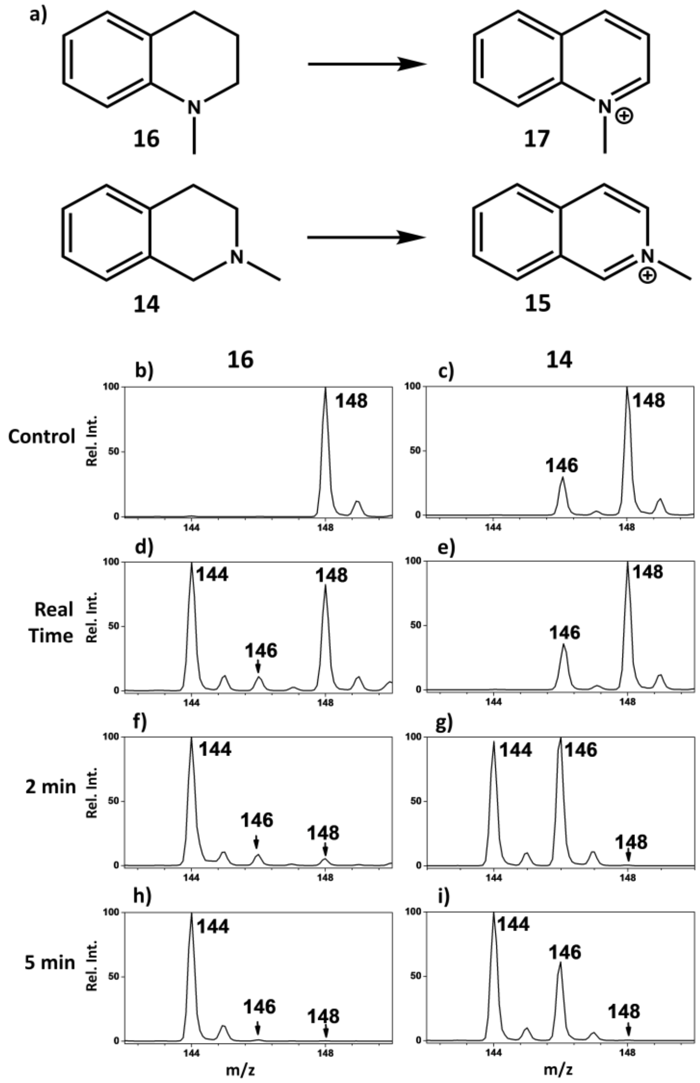

Figure S8: a) Schematic representing N-methyl-THQ **16** dehydrogenation produce N-methylquinolinum, **17** as the product and N-methyl-THiQ **14** dehydrogenation produces N-methylisoquinolinum, **15** as the product; Mass spectra showing analysis of dehydrogenation of **14** and **16** into **15** and **17** respectively using RPSP. The first row represent control, MS spectra for 100µM **14/16** and 5µM of Ru(bpy)32+ in ACN without irradiation. The second row represents real time, MS spectra recorder after simultaneous application of DC spray voltage and laser irradiation. The third and fourth rows represent MS spectra recorded after continuously irradiating the reaction mixture for 2min and 5min, respectively.

Topic 12: Reaction products and rates comparison of decahydroquinoline (DecaHQ, *MW* 139) and decahydroisoquinoline (DecaHiQ, *MW* 139) - Mass spectra for dehydrogenation by $Ru(bpy)_3^{2+}$ in bulk solutions. The decaHQ shows much faster dehydrogenation rates compared to decaHiQ. After 5 minutes of irradiation of the bulk solution I dehydrogenation has taken place in decaHQ and with the increase of reaction time we see a major product peak at *m/z* 170 which was identified by exact mass measurements using Orbitap to be molecular oxygen incorporated product. We assign this to the cycloaddition [2+2] of $^1O_2$ to the first dehydrogenated product. Similarly, for decaHiQ, with the increase of irradiation time produces a major product at *m/z* 307 which was identified by exact mass measurements to be molecular oxygen incorporated dimer using Orbitrap. With increased irradiation of decaHQ for 90 minutes we were able to identify 2nd dehydrogenation product at *m/z* 136.

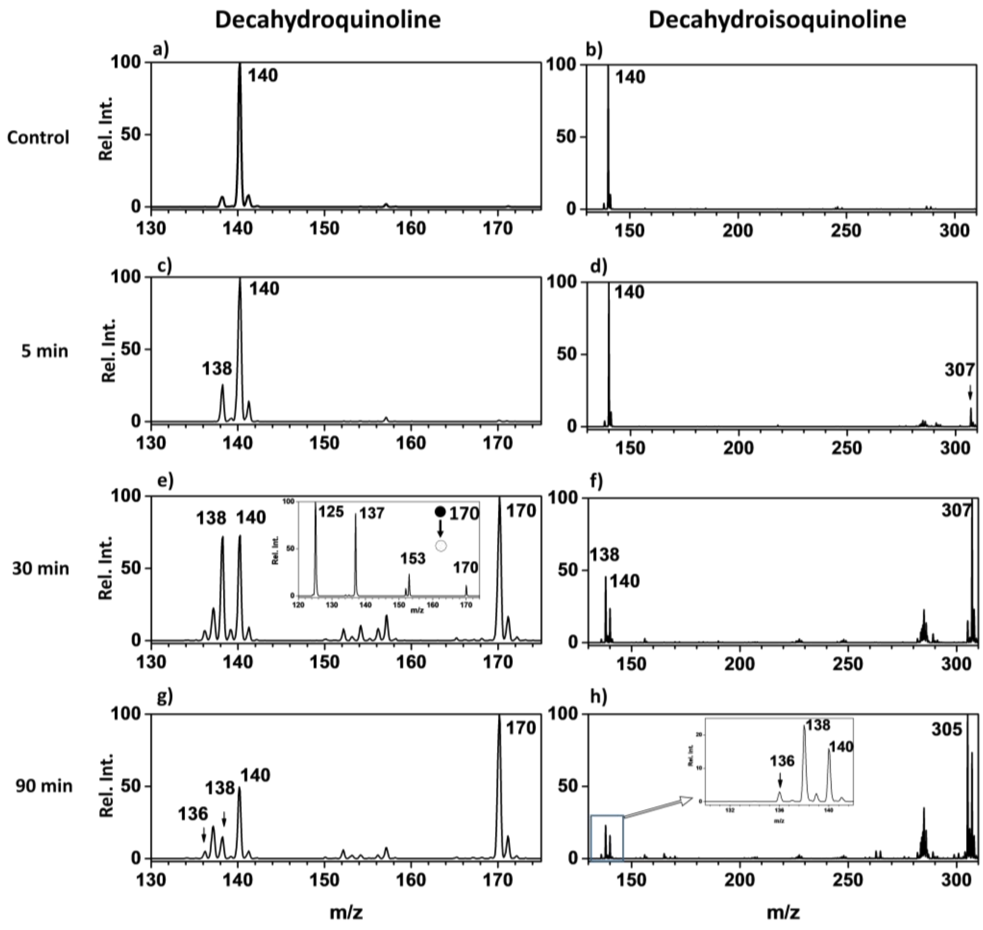

Figure S9: MS spectra showing 100µM of **DecaHQ/ DecaHiQ** reaction with 5µM $Ru(bpy)_3^{2+}$ in ACN bulk phase. The first row represent control, MS spectra for 100µM **DecaHQ/DecaHiQ** in ACN without irradiation. The second row represents 5 minutes, MS spectra recorder after 5minutes irradiation of the bulk solution with laser. The third and fourth rows represent MS spectra recorded after continuously irradiating of the bulk reaction mixture for 30min and 90min, respectively.

Topic 13: References

1. Chen, S., Wan, Q. & Badu-Tawiah, A. K. Picomole-Scale Real-Time Photoreaction Screening: Discovery of the Visible-Light-Promoted Dehydrogenation of Tetrahydroquinolines under Ambient Conditions. *Angew. Chemie - Int. Ed.* **55,** 9345–9349 (2016).

2. Wei, Z., Wleklinski, M., Ferreira, C. & Cooks, R. G. Reaction Acceleration in Thin Films with Continuous Product Deposition for Organic Synthesis. *Angew. Chemie - Int. Ed.* **56,** 9386–9390 (2017).

3. Jiang, J. X. *et al.* Conjugated microporous polymers with rose bengal dye for highly efficient heterogeneous organo-photocatalysis. *Macromolecules* **46,** 8779–8783 (2013).

4. Jiang, X. *et al.* A general method for N-methylation of amines and nitro compounds with dimethylsulfoxide. *Chem. - A Eur. J.* **20,** 58–63 (2014).

5. Van Haren, M. J. *et al.* A Rapid and Efficient Assay for the Characterization of Substrates and Inhibitors of Nicotinamide N-Methyltransferase. *Biochemistry* **55,** 5307–5315 (2016).

6. Ruiz, A., Rocca, P., Marsais, F., Godard, A. & Quéguiner, G. Pyridinium chloride: A new reagent for N-demethylation of N-methylazinium derivatives. *Tetrahedron Lett.* **38,** 6205–6208 (1997).

7. Maier, J. P. & Turner, D. W. Steric inhibition of resonance studied by molecular photoelectron spectroscopy. *J. Chem. Soc. Faraday Trans. 2* **69,** 521–531 (1973).

8. Morishima, I., Yoshikawa, K., Hashimoto, M. & Bekki, K. Homoallylic Interaction between the Nitrogen Lone Pair and the Nonadjacent π Bond in Cyclic and Bicyclic Amines. I. Photoelectron Spectroscopic Study. *J. Am. Chem. Soc.* **97,** 4283–4288 (1975).
